# Improved Prognostic Performance of Right Atrial Pressure-Corrected Cardiac Power Output in Pulmonary Hypertension and Heart Failure with Preserved Ejection Fraction

**DOI:** 10.1007/s12265-023-10429-y

**Published:** 2023-08-29

**Authors:** Yihang Wu, Pengchao Tian, Lin Liang, Yuyi Chen, Jiayu Feng, Boping Huang, Liyan Huang, Xuemei Zhao, Jing Wang, Jingyuan Guan, Xinqing Li, Jian Zhang, Yuhui Zhang

**Affiliations:** 1 Heart Failure Center, State Key Laboratory of Cardiovascular Disease, Fuwai Hospital, National Center for Cardiovascular Diseases, Chinese Academy of Medical Sciences & Peking Union Medical College, No. 167 Beilishi Road, Xicheng District, Beijing, 100037 China; 2 Key Laboratory of Clinical Research for Cardiovascular Medications, National Health Committee, Beijing, China

**Keywords:** Heart failure with preserved ejection fraction, Cardiac power output, Right atrial pressure, Right heart function, Prognosis

## Abstract

Cardiac power output (CPO) is a powerful predictor of adverse outcomes in heart failure (HF). However, the original formula of CPO included the difference between mean arterial pressure and right atrial pressure (RAP). The prognostic performance of RAP-corrected CPO (CPO_RAP_) remains unknown in heart failure with preserved ejection fraction (HFpEF). We studied 101 HF patients with a left ventricular ejection fraction > 40% who had pulmonary hypertension due to left heart disease. CPO_RAP_ was significantly more discriminating than CPO in predicting outcomes (Delong test, *P* = 0.004). Twenty-five (24.8%) patients presented with dis-concordantly high CPO_RAP_ and low CPO when stratified by the identified CPO_RAP_ threshold of 0.547 W and the accepted CPO threshold of 0.803 W. These patients had the lowest RAP, and their cumulative incidence was comparable with those with concordantly high CPO and CPO_RAP_ (*P* = 0.313). CPO_RAP_ might identify patients with right ventricular involvement, thereby providing better prognostic performance than CPO in HFpEF.

**Graphical Abstract:**

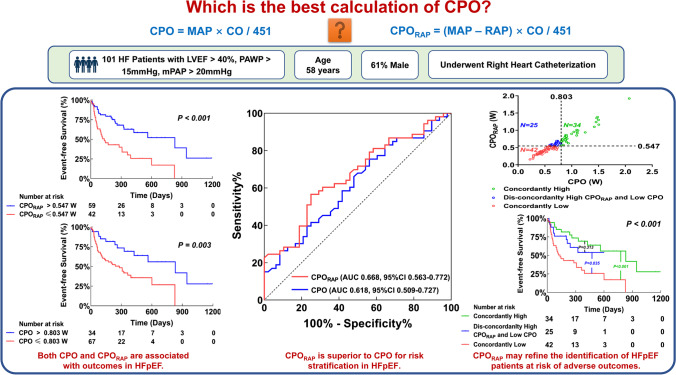

**Supplementary Information:**

The online version contains supplementary material available at 10.1007/s12265-023-10429-y.

## Introduction

The heart is a muscular pump supplying hydraulic energy to generate both flow (cardiac output [CO]) and pressure to maintain circulation. Cardiac power output (CPO), a measure of cardiac performance, is the product of simultaneously measured CO and mean arterial pressure (MAP) (namely, *CPO* = *MAP* × *CO* / 451) to express cardiac pump function [1]. Several studies have demonstrated that CPO is a powerful predictor of adverse clinical outcomes in heart failure with reduced ejection fraction (HFrEF) and cardiogenic shock [1–3]. The prognostic value of CPO measured by echocardiography has also been determined in patients with heart failure with preserved ejection fraction (HFpEF) [4]. However, the initial derivation of CPO by Tan included the difference between MAP and right atrial pressure (RAP) in the calculation, before multiplying by CO (namely, RAP-corrected CPO [CPO_RAP_] = [*MAP*-*RAP*] × *CO* / 451) [5]. The RAP component has been omitted in clinical practice and research to simplify the calculation over the past decade. Recently, the original formula has been revisited by Lim, noting the overestimation of CPO without the inclusion of RAP, particularly in patients with elevated intracardiac filling pressures [6]. Two subsequent studies have demonstrated that the prognostic performance of CPO_RAP_ is superior to CPO in both acute decompensated HFrEF and cardiogenic shock [7, 8]. However, the prognostic value of CPO_RAP_ in HFpEF remains unclear. In addition, few data regarding the prognostic impact of right heart catheterization (RHC)-derived CPO and CPO_RAP_ were available in HFpEF.

Accordingly, we investigated the association of CPO and CPO_RAP_ with clinical outcomes and hypothesized that CPO_RAP_ would provide better prognostic performance than CPO in the settings of HFpEF and heart failure with mildly reduced ejection fraction.

## Methods

### Study Population

In this retrospective cohort study, we enrolled consecutive heart failure (HF) patients aged ≥ 18 years with suspected pulmonary hypertension (PH) from November 2013 to June 2022. Patients underwent RHC at the Heart Failure Care Unit of our hospital. Patients were included if they (1) had pulmonary arterial wedge pressure (PAWP) > 15 mmHg; (2) had mean pulmonary arterial pressure (mPAP) > 20 mmHg; (3) had left ventricular ejection fraction (LVEF) > 40% by echocardiogram (calculated by modified Simpson method); (4) had no evidence of congenital heart disease, intracardiac shunts, or moderate to severe valvular disease. Patients with other subtypes of PH (groups 1, 3, 4, and 5) were excluded [9]. All patients completed blood tests and echocardiography within 24 h after undergoing RHC. Data regarding demographics, relevant cardiovascular and comorbid conditions, HF therapies, and laboratory and echocardiographic tests were collected by qualified cardiologists. The patients were followed up by telephone or clinic visits. Clinical outcomes including death and HF rehospitalization were collected. None of the patients underwent heart transplantation during the follow-up period. The primary outcome was event-free survival. This study complied with the principles outlined in the Declaration of Helsinki and was approved by the ethics committee of our hospital. Written informed consent was obtained from all participants.

### Right Heart Catheterization and Hemodynamic Assessment

RHC was performed using the Swan-Ganz catheter (Edwards Lifesciences, USA) with echocardiographic and pressure waveform guidance. After minimal sedation, echocardiography-guided catheterization was performed through the right internal jugular vein to the pulmonary artery by HF specialists. The external pressure transducer was zeroed at the mid-thoracic level in each patient, and all pressure tracings were continuously recorded and stored. Pressure measurements were recorded at end-expiration during spontaneous breathing. Cardiac output (CO) was measured using the thermodilution method. Key hemodynamic measures recorded at the time of RHC included heart rate, systolic/diastolic/mean arterial pressure (SAP/DAP/MAP), RAP, systolic/diastolic/mean pulmonary arterial pressure (s/d/mPAP), PAWP, stroke volume (SV), and CO. Systemic vascular resistance (SVR) was calculated in Wood units as (*MAP* − *RAP*) / *CO*. Left ventricular effective arterial elastance (LV-Ea) was calculated as 0.9 × *SAP* / *SV*. Pulmonary vascular resistance (PVR) was calculated in Wood units as (*mPAP* − *PAWP*) / *CO*. Pulmonary arterial compliance (PAC) was estimated as *SV* / (*sPAP* − *dPAP*). CPO was defined in Watt (W) units as *MAP* × *CO* / 451, and CPO_RAP_ was defined as (*MAP*-*RAP*) × *CO* / 451. Pulmonary artery pulsatility index was calculated as (*sPAP*-*dPAP*) / *RAP*.

### Statistical Analysis

Categorical values were expressed as absolute numbers (percentage) and continuous variables as median (interquartile range) or mean ± standard deviation. The Shapiro-Wilk test was used to assess normality. Differences were evaluated for continuous variables by one-way analysis of variance if normally distributed, or the Mann-Whitney U test as well as the Kruskal–Wallis test if non-normally distributed, and for categorical variables using Pearson’s *χ*^2^ test or Fisher’s exact test. The receiver operating characteristic (ROC) analysis was used to calculate a precise cut-off of CPO_RAP_ (0.547 W) and CPO (0.803 W) that would best discriminate event-free survival. We assessed the ability of CPO_RAP_ and CPO to discriminate between patients who had reached the primary outcome and those who were event-free by the close of follow-up by calculating the area under the curve (AUC), and compared performance using the Delong method. In the outcome analysis, age, sex, body mass index (BMI), New York Heart Association (NYHA) functional class, LVEF, tricuspid annular plane systolic excursion (TAPSE), N-terminal pro-B-type natriuretic peptide (NT-proBNP), history of coronary artery disease, atrial fibrillation, hypertension, diabetes, hyperlipidemia, use of loop diuretic, and hemodynamic variables were selected as possible confounders of the CPO_RAP_ association and were assessed in the univariate model. The variables that remained significant at the 0.10 level in univariable analysis were considered for inclusion in the multivariate model. A forward stepwise method was used to remove variables with a *P* value > 0.10 and enter variables that met a 0.05 significance level for the selection of the final multivariate model. The Kaplan-Meier analysis was used to compare the different groups for the estimation of outcomes with the log-rank test. Two-sided *P* values of < 0.05 were considered statistically significant. SPSS Statistics 26 (IBM, USA), R version 4.0.2 (The R Foundation, Austria), and Prism 8 (GraphPad Software, USA) were used for statistical analyses.

## Results

### Clinical Characteristics

**Fig. 1:**
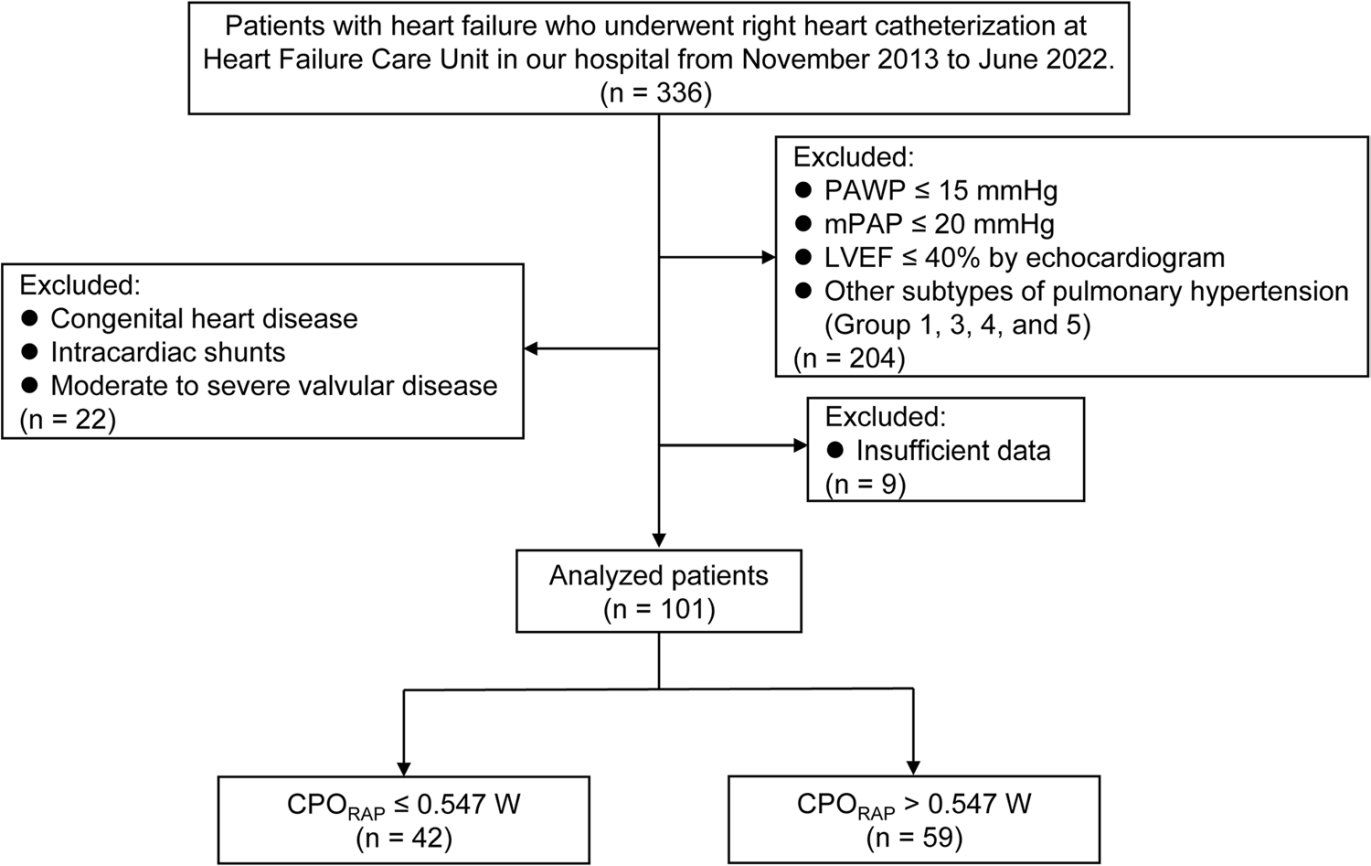
Flow chart of subject selection and analysis. CPO, cardiac power output; CPO_RAP_, right atrial pressure-corrected cardiac power output; LVEF, left ventricular ejection fraction; mPAP, mean pulmonary arterial pressure; PAWP, pulmonary arterial wedge pressure; RAP, right atrial pressure

We identified 336 HF patients who underwent RHC between November 2013 and June 2022. After the screening, 101 patients met the inclusion criterion and were finally included in the analysis (Fig. 1). The median age was 58 (48–66) years and about 61% were male (Table 1). Age, sex, BMI, comorbidities, and medications did not differ between the two groups (all *P* > 0.05). Regarding laboratory tests and echocardiography, patients with *CPO*_*RAP*_ ≤ 0.547 W had higher serum NT-proBNP values (*P* = 0.001), lower LVEF (*P* = 0.007), and lower TAPSE (*P* < 0.001). As expected, patients with lower CPO_RAP_ had lower CO and SV, lower SAP and MAP, higher SVR and LV-Ea, higher mPAP and dPAP, higher PAWP, higher PVR, and lower PAC (all *P* < 0.05).

**Table 1:** Clinical and hemodynamic characteristics stratified by CPO_RAP_

	All (*n* = 101)	CPO_RAP_ ≤ 0.547 (*n* = 42)	CPO_RAP_ > 0.547 (*n* = 59)	*P* value
Clinical characteristics				
Age (y)	58 (48–66)	60 (46–69)	58 (49–65)	0.992
Men, *n* (%)	62 (61.4%)	27 (64.3%)	35 (59.3%)	0.614
BMI (kg/m^2^)	22.9 (20.6–25.6)	22.5 (20.6–24.2)	23.3 (20.7–26.1)	0.101
Coronary artery disease, *n* (%)	24 (23.8%)	9 (21.4%)	15 (25.4%)	0.642
Atrial fibrillation, *n* (%)	39 (38.6%)	19 (45.2%)	20 (33.9%)	0.249
Hypertension, *n* (%)	31 (30.7%)	9 (21.4%)	22 (37.3%)	0.089
Diabetes, *n* (%)	16 (15.8%)	6 (14.3%)	10 (16.9%)	0.718
Hyperlipidemia, *n* (%)	22 (21.8%)	7 (16.7%)	15 (25.4%)	0.293
NYHA functional class				0.042
II, *n* (%)	21 (20.8%)	4 (9.5%)	17 (28.8%)	
III, *n* (%)	38 (37.6%)	20 (47.6%)	18 (30.5%)	
IV, *n* (%)	42 (41.6%)	18 (42.9%)	24 (40.7%)	
Medications				
Beta blocker, *n* (%)	64 (63.4%)	26 (61.9%)	38 (64.4%)	0.797
ACEI /ARB/ARNI, *n* (%)	23 (22.8%)	6 (14.3%)	17 (28.8%)	0.086
MRA, *n* (%)	64 (63.4%)	27 (64.3%)	37 (62.7%)	0.871
Loop diuretic, *n* (%)	91 (90.1%)	42 (100.0%)	49 (83.1%)	0.013
Laboratory values				
Hemoglobin (g/l)	128 ± 22	126 ± 22	129 ± 22	0.607
Creatinine (μmol/l)	92 (71–112)	93 (70–118)	91 (71–108)	0.725
NT-proBNP (pg/ml)	3877 (1382–7762)	6299 (2690–11,975)	2307 (979–5430)	0.001
Echocardiography				
LVEF (%)	55 (46–60)	49 (45–60)	57 (50–62)	0.007
Right ventricular dimension (mm)	26 (22–30)	26 (22–30)	26 (23–30)	0.425
TAPSE (mm)	17 (13–19)	15 (12–17)	18 (16–21)	< 0.001
Hemodynamics				
CO (l/min)	4.1 (3.1–4.5)	2.9 (2.3–3.4)	4.3 (4.1–5.2)	< 0.001
SV (ml)	57 (37–69)	34 (30–52)	62 (52–78)	< 0.001
HR (bpm)	75 ± 13	77 ± 15	73 ± 12	0.201
SAP (mmHg)	101 (95–114)	97 (93–103)	107 (97–124)	< 0.001
MAP (mmHg)	79 (75–86)	77 (72–81)	81 (76–93)	0.001
DAP (mmHg)	67 (62–74)	67 (62–72)	68 (62–78)	0.067
SVR (Wood)	17.4 (13.7–23.0)	22.8 (17.3–27.5)	16.0 (12.7–18.2)	< 0.001
LV-Ea (mmHg/ml)	1.7 (1.4–2.6)	2.6 (1.7–3.2)	1.5 (1.3–1.8)	< 0.001
RAP (mmHg)	12 (7–17)	14 (7–20)	11 (7–15)	0.071
sPAP (mmHg)	44 (35–54)	49 (36–60)	43 (34–50)	0.076
mPAP (mmHg)	30 (24–38)	33 (27–42)	29 (23–36)	0.044
dPAP (mmHg)	22 (17–28)	24 (20–30)	21 (16–26)	0.020
PAWP (mmHg)	22 (17–27)	24 (19–28)	21 (16–24)	0.027
PVR (Wood)	2.2 (1.3–3.4)	3.0 (2.2–4.7)	1.6 (1.1–2.6)	< 0.001
PAC (ml/mmHg)	2.4 (1.5–3.9)	1.5 (1.1–2.6)	3.3 (2.3–4.5)	< 0.001
PAPi	1.9 (1.2–2.9)	1.8 (1.1–2.9)	2.0 (1.3–3.0)	0.486
CPO (W)	0.700 (0.541–0.873)	0.498 (0.394–0.605)	0.822 (0.717–0.998)	< 0.001
CPO_RAP_ (W)	0.576 (0.439–0.718)	0.412 (0.325–0.493)	0.688 (0.600–0.864)	< 0.001

*ACEI*, angiotensin-converting enzyme inhibitor; *ARB*, angiotensin-receptor blocker; *ARNI*, angiotensin receptor-neprilysin inhibitor; *BMI*, body mass index; *CO*, cardiac output; *CPO*, cardiac power output; *CPO*_*RAP*_, right atrial pressure-corrected cardiac power output; *DAP*, diastolic arterial pressure; *dPAP*, diastolic pulmonary arterial pressure; *HR*, heart rate; *LV-Ea*, left ventricular effective arterial elastance; *LVEF*, left ventricular ejection fraction; *NYHA*, New York Heart Association; *MAP*, mean arterial pressure; *mPAP*, mean pulmonary arterial pressure; *MRA*, mineralocorticoid receptor antagonist; *NT-proBNP*, N-terminal pro-B-type natriuretic peptide; *PAC*, pulmonary arterial compliance; *PAPi*, pulmonary artery pulsatility index; *PAWP*, pulmonary arterial wedge pressure; *PVR*, pulmonary vascular resistance; *RAP*, right atrial pressure; *SAP*, systolic arterial pressure; *sPAP*, systolic pulmonary arterial pressure; *SV*, stroke volume; *SVR*, systemic vascular resistance; *TAPSE*, tricuspid annular plane systolic excursion

### Clinical Outcomes Associated with CPO and CPORAP

**Fig. 2:**
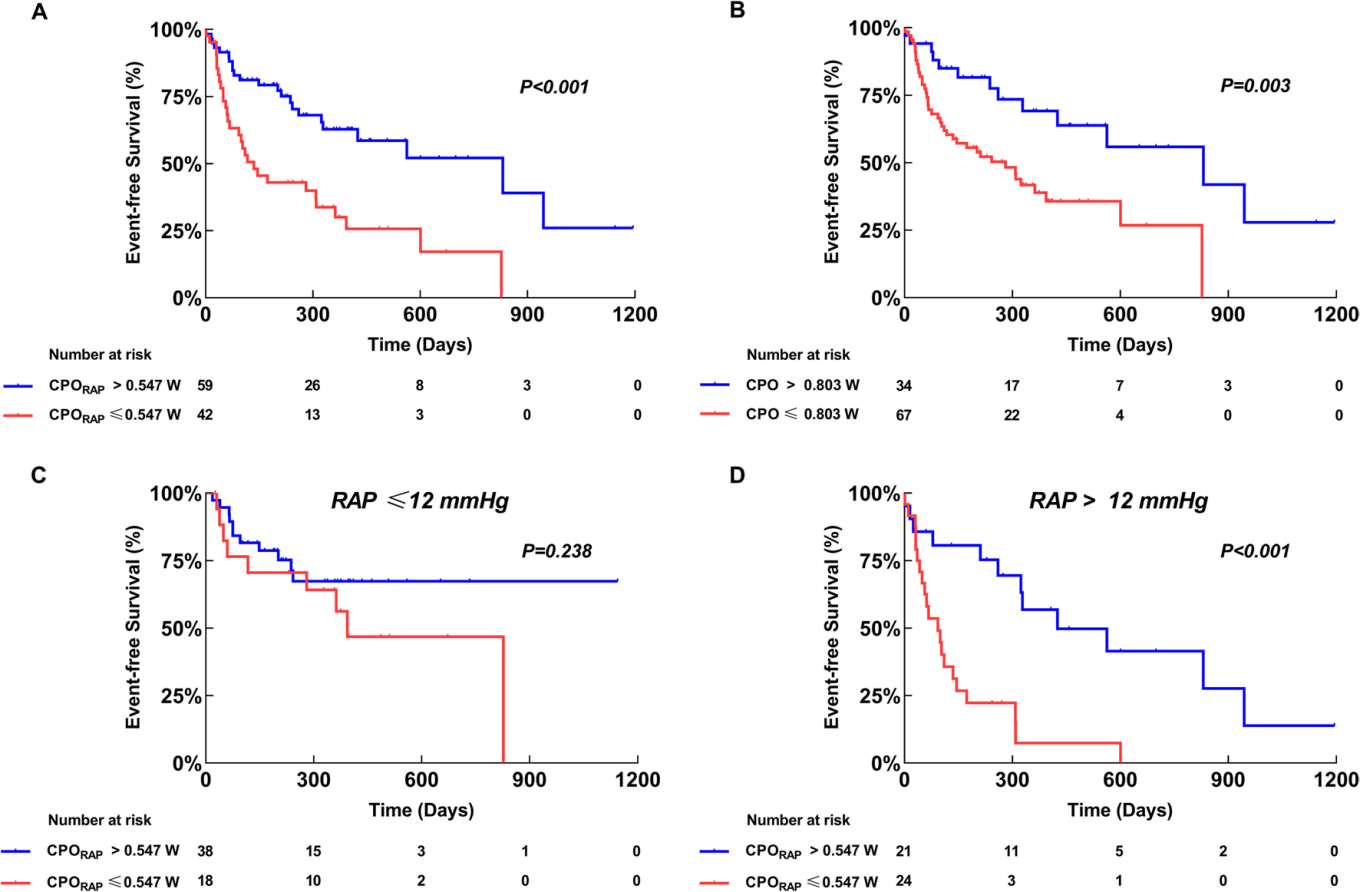
Survival Analysis. The Kaplan-Meier estimates of time to event-free survival stratified by CPO_RAP_ for the full cohort (**A**), stratified by CPO for the full cohort (**B**), stratified by CPO_RAP_ for patients with right atrial pressure ≤ 12 mmHg (**C**), stratified by CPO_RAP_ for patients with right atrial pressure > 12 mmHg (**D**). CPO, cardiac power output; CPO_RAP_, right atrial pressure-corrected cardiac power output; RAP, right atrial pressure

The median duration of the follow-up period was 327 days (139–522). During the follow-up, 14 (13.9%) patients died, and 39 (38.6%) patients were rehospitalized for HF. In univariable Cox regression analysis, CPO_RAP_ was independently associated with event-free survival (HR 0.102, 95% confidence interval [CI] 0.027–0.391). After multivariate adjustment, CPO_RAP_ remained significantly associated with the primary outcome (HR 0.211, 95% CI 0.052–0.864) (Supplemental Table 1). CPO was also independently associated with event-free survival in univariable analysis (HR 0.219, 95% CI 0.075–0.644), and remained significantly associated with the primary outcome in the adjusted analyses (HR 0.270, 95% CI 0.083–0.880) (Supplemental Table 2). The Kaplan-Meier analysis and log-rank test revealed significant differences in event-free survival, whether using the optimal cut-off of 0.547 W for CPO_RAP_ (*P* < 0.001) or 0.803 W for CPO (*P* < 0.003). When further analyzing CPO_RAP_ by RAP above or below the median (12 mmHg), a significant difference in the outcome was only found for patients with RAP of more than 12 mmHg (*P* < 0.001) (Fig. 2). In addition, a significant difference in the outcome was also only found for patients with PVR of more than 2.2 WU (*P* = 0.026). However, the difference in the outcome was significant regardless of analyzing CPO_RAP_ by mPAP above or below the median (30 mmHg) (all *P* < 0.05) (Supplemental Fig. 1).

**Fig. 3:**
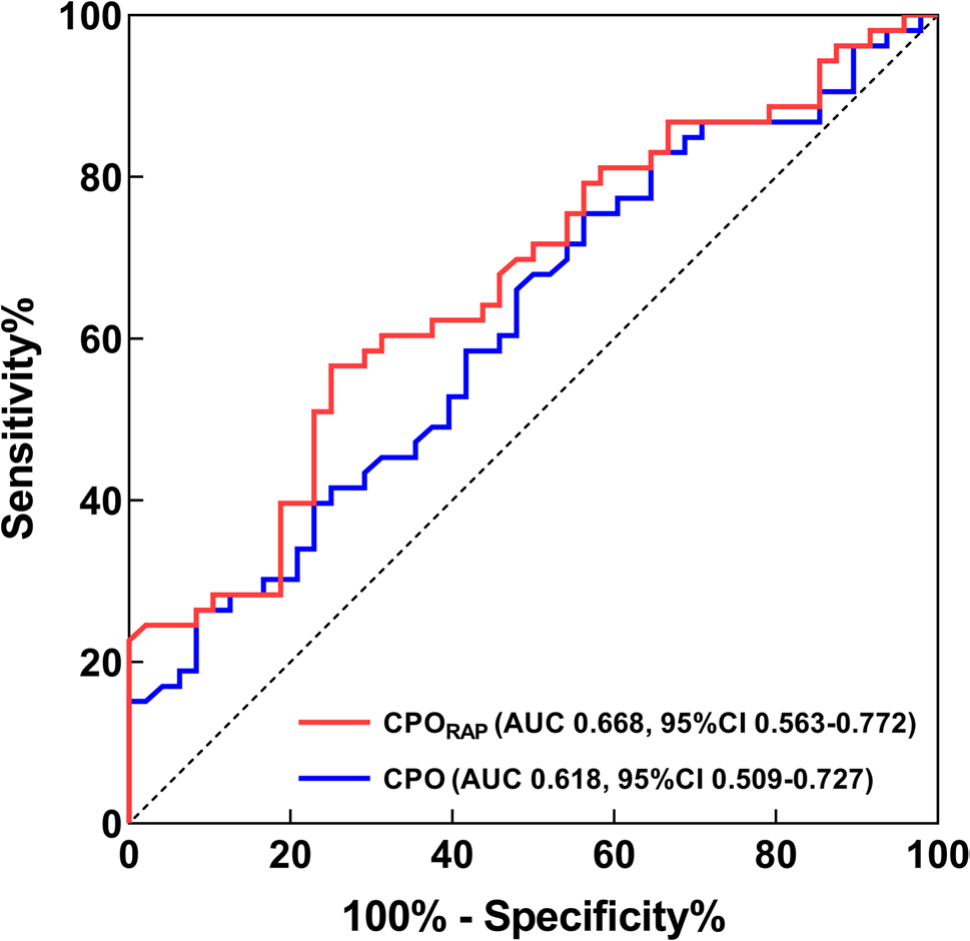
Receiver operating characteristic curves for prediction of event-free survival. CPO, cardiac power output; CPO_RAP_, right atrial pressure-corrected cardiac power output

Based on ROC analysis, CPO_RAP_ was significantly more discriminating than CPO for the prediction of event-free survival, with an AUC of 0.668 for CPO_RAP_ (95% CI: 0.563–0.772) and 0.618 for CPO (95% CI: 0.509–0.727) (Delong test, CPO_RAP_ vs. CPO: *P* = 0.004) (Fig. 3).

### Reclassification Analyses

**Fig. 4:**
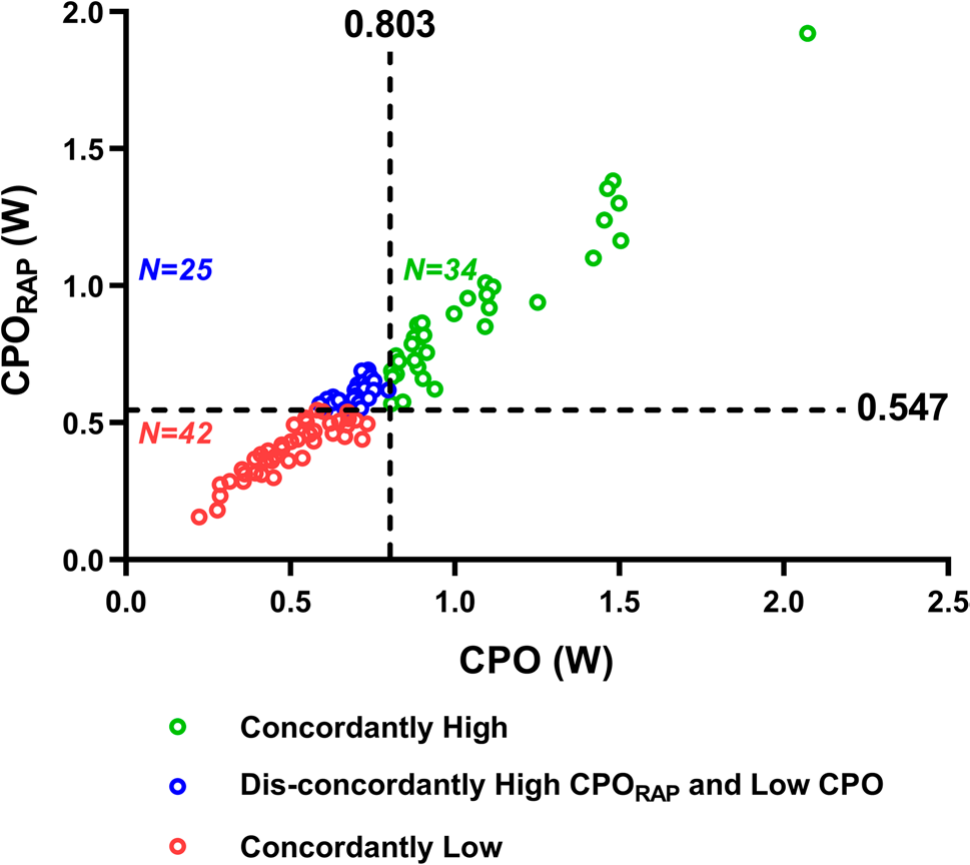
Distribution of CPO_RAP_ and CPO. CPO, cardiac power output; CPO_RAP_, right atrial pressure-corrected cardiac power output

We further investigated the impact of reclassification by the identified CPO_RAP_ threshold of 0.547 W compared to the accepted CPO threshold of 0.803 W. A total of 42 (41.6%) patients presented with concordantly low CPO_RAP_ and CPO, 34 (33.7%) patients presented with concordantly high CPO_RAP_ and CPO, and 25 (24.8%) patients presented with dis-concordantly high CPO_RAP_ and low CPO (Fig. 4). Clinical characteristics and hemodynamic profiles for the three groups defined according to CPO and CPO_RAP_ agreement are described in Table 2. Patients in the discordant group showed intermediate serum NT-proBNP values and LVEF between patients in the concordantly high and low groups. As expected, they also exhibited intermediate CO and SV, but not MAP. Compared to the other two groups, patients in the discordant group had the lowest RAP and the highest TAPSE. In addition, there were significant differences in TAPSE, RAP, SVR, LV-Ea, PVR, and PAC between the discordant group and the concordantly low group (all *P* < 0.05), but there was no statistical difference in the above parameters between the discordant group and the concordantly high group (all *P* > 0.05).

**Table 2:** Clinical and hemodynamic characteristics stratified by CPO_RAP_ and CPO agreement groups

	Concordantly low (*n* = 42)	Disconcordant (*n* = 25)	Concordantly high (*n* = 34)	*P* value
Clinical characteristics				
Age (y)	60 (46–69)	54 (46–66)	60 (53–65)	0.568
Men, *n* (%)	27 (64.3%)	15 (60.0%)	20 (58.8%)	0.877
BMI (kg/m^2^)	22.5 (20.6–24.2)	22.1 (19.7–26.1)	23.5 (22.0–26.9)	0.110
Coronary artery disease, *n* (%)	9 (21.4%)	5 (20.0%)	10 (29.4%)	0.631
Atrial fibrillation, *n* (%)	19 (45.2%)	6 (24.0%)	14 (41.2%)	0.210
Hypertension, *n* (%)	9 (21.4%)	5 (20.0%)	17 (50.0%)	0.011
Diabetes, *n* (%)	6 (14.3%)	3 (12.0%)	7 (20.6%)	0.652
Hyperlipidemia, *n* (%)	7 (16.7%)	5 (20.0%)	10 (29.4%)	0.396
NYHA functional class				0.074
II, *n* (%)	4 (9.5%)	5 (20.0%)	12 (35.3%)	
III, *n* (%)	20 (47.6%)	8 (32.0%)	10 (29.4%)	
IV, *n* (%)	18 (42.9%)	12 (48.0%)	12 (35.3%)	
Medications				
Beta blocker, *n* (%)	26 (61.9%)	14 (56.0%)	24 (70.6%)	0.500
ACEI /ARB/ARNI, *n* (%)	6 (14.3%)	7 (28.0%)	10 (29.4%)	0.228
MRA, *n* (%)	27 (64.3%)	16 (64.0%)	21 (61.8%)	0.972
Loop diuretic, *n* (%)	42 (100.0%)	21 (84.0%)	28 (82.4%)	0.007
Laboratory values				
Hemoglobin (g/l)	126 ± 22	129 ± 23	129 ± 21	0.876
Creatinine (μmol/l)	93 (70–118)	80 (64–99)	95 (80–111)	0.152
NT-proBNP (pg/ml)	6299 (2690–11,975)	4229 (1442–8643)	1648 (518–4362)^a^	< 0.001
Echocardiography				
LVEF (%)	49 (45–60)	55 (47–63)	57 (53–61)^a^	0.023
Right ventricular dimension (mm)	26 (22–30)	24 (23–28)	28 (25–34)^b^	0.018
TAPSE (mm)	15 (12–17)	19 (17–21)^a^	17 (13–21)^a^	< 0.001
Hemodynamics				
CO (l/min)	2.9 (2.3–3.4)	4.2 (3.8–4.3)^a^	4.9 (4.3–6.5)^a,b^	< 0.001
SV (ml)	34 (30–52)	58 (47–62)^a^	71 (60–84)^a,b^	< 0.001
HR (bpm)	77 ± 15	74 ± 12	73 ± 11	0.420
SAP (mmHg)	97 (93–103)	98 (95–108)	120 (104–128)^a,b^	< 0.001
MAP (mmHg)	79 (72–81)	77 (74–80)	89 (79–98)^a,b^	< 0.001
DAP (mmHg)	67 (62–72)	66 (62–69)	74 (64–84)^a,b^	0.004
SVR (Wood)	22.8 (17.3–27.5)	16.5 (15.1–17.8)^a^	14.7 (11.1–19.8)^a^	< 0.001
LV-Ea (mmHg/ml)	2.6 (1.7–3.2)	1.7 (1.4–1.9)^a^	1.5 (1.2–1.8)^a^	< 0.001
RAP (mmHg)	14 (7–20)	8 (6–13)^a^	12 (9–17)	0.013
sPAP (mmHg)	49 (36–60)	44 (31–50)	43 (35–52)	0.187
mPAP (mmHg)	33 (27–42)	28 (23–36)	30 (24–36)	0.122
dPAP (mmHg)	24 (20–30)	19 (15–28)	21 (16–25)	0.062
PAWP (mmHg)	24 (19–28)	21 (17–27)	19 (16–24)	0.078
PVR (Wood)	3.0 (2.2–4.7)	1.8 (1.2–2.6)^a^	1.5 (1.1–2.8)^a^	< 0.001
PAC (ml/mmHg)	1.5 (1.1–2.6)	2.8 (2.3–3.8)^a^	3.7 (2.2–4.6)^a^	< 0.001
PAPi	1.8 (1.1–2.9)	2.1 (1.3–3.6)	1.8 (1.1–2.7)	0.271
CPO (W)	0.498 (0.394–0.605)	0.709 (0.640–0.737)^a^	0.910 (0.863–1.150)^a,b^	< 0.001
CPO_RAP_ (W)	0.412 (0.325–0.493)	0.600 (0.573–0.644)^a^	0.836 (0.710–1.000)^a,b^	< 0.001

*ACEI*, angiotensin-converting enzyme inhibitor; *ARB*, angiotensin-receptor blocker; *ARNI*, angiotensin receptor-neprilysin inhibitor; *BMI*, body mass index; *CO*, cardiac output; *CPO*, cardiac power output; *CPO*_*RAP*_, right atrial pressure-corrected cardiac power output; *DAP*, diastolic arterial pressure; *dPAP*, diastolic pulmonary arterial pressure; *HR*, heart rate; *LV-Ea*, left ventricular effective arterial elastance; *LVEF*, left ventricular ejection fraction; *NYHA*, New York Heart Association; *MAP*, mean arterial pressure; *mPAP*, mean pulmonary arterial pressure; *MRA*, mineralocorticoid receptor antagonist; *NT-proBNP*, N-terminal pro-B-type natriuretic peptide; *PAC*, pulmonary arterial compliance; *PAPi*, pulmonary artery pulsatility index; *PAWP*, pulmonary arterial wedge pressure; *PVR*, pulmonary vascular resistance; *RAP*, right atrial pressure; *SAP*, systolic arterial pressure; *sPAP*, systolic pulmonary arterial pressure; *SV*, stroke volume; *SVR*, systemic vascular resistance; *TAPSE*, tricuspid annular plane systolic excursion

^a^*P* < 0.05 vs. concordantly low group

^b^*P* < 0.05 vs. disconcordant group

**Fig. 5:**
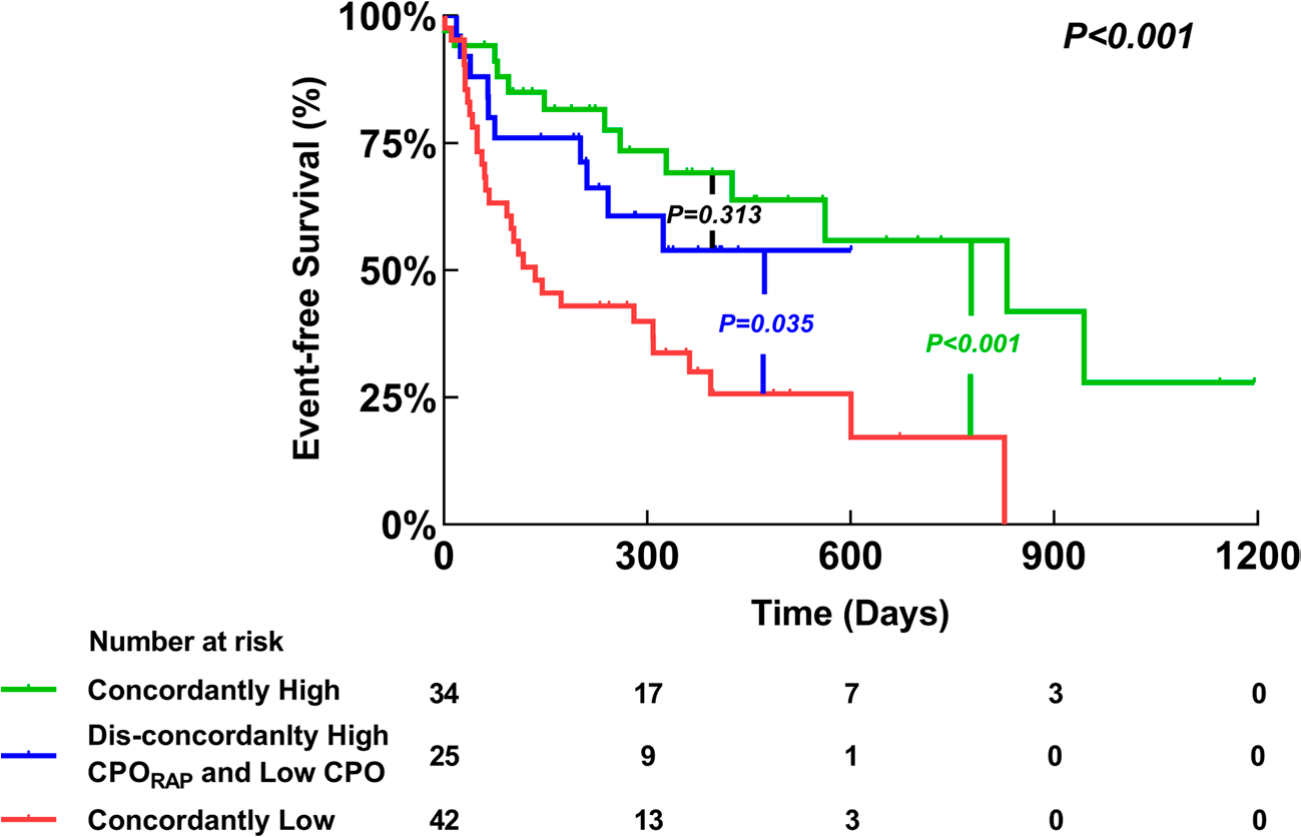
Survival analysis. The Kaplan-Meier estimates of time to event-free survival stratified by CPO_RAP_ and CPO agreement

Patients with concordantly low CPO_RAP_ and CPO had a significantly worse outcome than those with concordantly high CPO_RAP_ and CPO, as well as those with dis-concordantly high CPO_RAP_ and low CPO (all *P* < 0.05). There was no significant difference in the outcome between patients with concordantly high CPO_RAP_ and CPO and those with dis-concordantly high CPO_RAP_ and low CPO (*P* = 0.313) (Fig. 5).

## Discussion

To our knowledge, this is the first report to explore the prognostic value of RHC-derived CPO and CPO_RAP_ in HF patients with an LVEF > 40%. The data in the present study demonstrate that (1) both CPO and CPO_RAP_ were associated with adverse outcomes; (2) CPO_RAP_ was superior to CPO for risk stratification; (3) the cumulative incidence of patients with low CPO reclassified as high CPO_RAP_ was comparable with that of patients with concordantly high CPO and CPO_RAP_.

CPO is a comprehensive indicator of cardiac pump efficiency, and its prognostic effect has been well studied in patients with HF, despite the calculation of CPO in most previous studies did not incorporate RAP [1–3]. Since RAP is much lower than MAP in healthy people, the omission of RAP may not affect CPO calculation. However, in keeping with the concept of pressure–volume loop and Guytonian depictions of the circulatory system, RAP is an indispensable component of CPO calculation, especially when RAP is significantly elevated relative to MAP. Although the elevation in left ventricular end-diastolic pressure secondary to diastolic dysfunction is the main pathophysiological characteristic in HFpEF, the increase in RAP is also a relatively common hemodynamic profile in some patients [10, 11]. In our cohort of HF patients with an LVEF > 40%, all patients had hemodynamically defined PH, with a median mPAP of 30 mmHg and a median RAP of 12 mmHg. We demonstrated that both CPO and CPO_RAP_ were associated with adverse outcomes. These results were consistent with previous studies in patients with HFrEF or cardiogenic shock [7, 8]. Therefore, we extended on the previous studies and further found for the first time that CPO_RAP_ outperformed CPO in distinguishing patients who would experience adverse outcomes in HFpEF.

HFpEF accounts for more than half of patients with HF and frequently is associated with PH [12]. The elevation in left ventricular end-diastolic pressure and left atrial pressure are the triggers for the development of PH in HFpEF [13]. Secondary PH and pulmonary vascular disease may enhance right ventricular afterload, subsequently contributing to right ventricular dysfunction and remodeling, leading to a further increase in RAP [14, 15]. Previous studies have demonstrated that RAP could represent the cumulative cardiac burden in HFpEF [16, 17], and higher RAP is independently associated with adverse outcomes in HFpEF [17, 18]. Therefore, compared with CPO, CPO_RAP_ integrates an additional risk factor and could better identify patients with predominantly right ventricular or biventricular involvement, which could be an explanation for the better prognostic performance for adverse outcomes of CPO_RAP_ than CPO. In the present study, there was a significant difference in the outcome in patients with RAP of more than 12 mmHg after stratified by the cut-off of CPO_RAP_, whereas patients with RAP of 12 mmHg or less were not. In addition, patients in the dis-concordantly high CPO_RAP_ and low CPO group had higher TAPSE, higher CO and SV, higher PAC, lower RAP, lower SVR and LV-Ea, and lower PVR compared with patients in the concordantly low group. These are all established markers reflective of right heart function, cardiac performance, or ventricular afterload, which may partly explain why the cumulative incidence of patients in the discordant group was significantly lower than those in the concordantly low group. Taken together, CPO_RAP_ incorporates four fundamental hemodynamic parameters (SV, heart rate, MAP, and RAP) and considers both cardiac pump function and right heart function, making it superior to CPO in risk stratification.

Indeed, HFpEF patients without right heart dysfunction can be well evaluated by the established CPO calculation. However, it is now increasingly recognized that right heart dysfunction is prevalent and contributes importantly to poor prognosis in HFpEF [19]. Moreover, several studies have identified intracardiac pressures as powerful predictors of adverse outcomes in HF [20, 21]. It is obvious that the inclusion of filling pressure into measures of cardiac function could improve prognostic performance. Therefore, compared with CPO, CPO_RAP_ could be a more comprehensive index of the global performance of the heart in HFpEF. More importantly, CPO_RAP_ could also be measured and calculated by echocardiography, as RAP could be readily estimated based on inferior vena cava diameter and its respiratory changes. Future studies are needed to validate the prognostic performance of echocardiography-derived CPO_RAP_ in HFpEF and explored whether CPO_RAP_ could be used as an indicator to evaluate the therapeutic efficacy of HFpEF.

Overall, compared with CPO, CPO_RAP_ may refine the identification of HFpEF patients at risk of adverse outcomes. Nevertheless, this study does not undermine previous reports on the predictive value of CPO in its current widely used calculations. The present study reemphasizes the concept of CPO and calls for further utilization and validation of its original derivation (CPO_RAP_) in more clinical studies, especially with the increasing importance of right heart function in the assessment of HFpEF [22].

### Limitations

Several limitations in the present study should be noted. First, this is a retrospective, single-center study with a relatively small number of patients in our cohort. However, we tried our best to ensure the accuracy of the available data. In addition, the study results are consistent with previous studies in patients with HFrEF and are supported by pathophysiological rationale. Second, all HFpEF patients in our cohort had PH. Considering that patients with PH are more likely to present with right heart dysfunction and elevated RAP, selection bias might exist in our research. Third, RHC is not a necessary diagnostic procedure for HFpEF, especially in those patients who do not have suspected PH or who have already been diagnosed with HFpEF by routine examination. The results may, therefore, not apply to the whole HFpEF population.

## Conclusion

Both CPO and CPO_RAP_ are associated with adverse outcomes in patients with HFpEF. By incorporating RAP, CPO_RAP_ integrates both cardiac performance and right heart function and could better reflect the true cardiac pump ability in HFpEF. Compared with CPO, CPO_RAP_ could enhance the prognostic value. Our data may provide new insights into the assessment of patients with HFpEF, especially those with suspected right heart involvement.

### Supplementary Information

Below is the link to the electronic supplementary material.

Supplementary file1 (PDF 293 KB)

## Data Availability

The data will be shared on reasonable request to the corresponding author.

## Abbreviations

AUC: Area under the curve
BMI: Body mass index
CO: Cardiac output
CPO: Cardiac power output
CPO_RAP_: Right atrial pressure-corrected cardiac power output
DAP: Diastolic arterial pressure
dPAP: Diastolic pulmonary arterial pressure
HF: Heart failure
HFpEF: Heart failure with preserved ejection fraction
HFrEF: Heart failure with reduced ejection fraction
LV-Ea: Left ventricular effective arterial elastance
LVEF: Left ventricular ejection fraction
MAP: Mean arterial pressure
mPAP: Mean pulmonary arterial pressure
NT-proBNP: N-terminal pro-B-type natriuretic peptide
NYHA: New York Heart Association
PAWP: Pulmonary arterial wedge pressure
PAC: Pulmonary arterial compliance
PH: Pulmonary hypertension
PVR: Pulmonary vascular resistance
RAP: Right atrial pressure
ROC: Receiver operating characteristic
RHC: Right heart catheterization
SAP: Systolic arterial pressure
sPAP: Systolic pulmonary arterial pressure
SV: Stroke volume
SVR: Systemic vascular resistance
TAPSE: Tricuspid annular plane systolic excursion
