# Supplementary material for: Improved Prognostic Performance of Right Atrial Pressure-Corrected Cardiac Power Output in Pulmonary Hypertension and Heart Failure with Preserved Ejection Fraction
Source: J Cardiovasc Transl Res. 2023 Aug 29;17(2):448–57. doi: 10.1007/s12265-023-10429-y (PMC11052873; doi:10.1007/s12265-023-10429-y)
Supplement: Supplementary file 1 — Supplementary file1 (PDF 293 KB) [file 12265_2023_10429_MOESM1_ESM.pdf]

SUPPLEMENTAL MATERIALS

Supplemental Table 1. Univariate and multivariate Cox regression models for event-free survival.

	Univariate Cox Regression		Multivariate Cox Regression	
	**HR (95%CI)**	**P value**	**HR (95%CI)**	**P value**
Age, y	0.992 (0.975-1.010)	0.396		
Sex, Male vs. Female	1.127 (0.646-1.967)	0.673		
BMI, kg/m^2	**0.928 (0.856-1.007)**	**0.074**	**-**	
NYHA functional class, Ⅳ vs. Ⅱ+Ⅲ	**2.062 (1.165-3.650)**	**0.013**	**-**	
LVEF, %	0.982 (0.951-1.014)	0.258		
TAPSE, mm	**0.948 (0.890-1.010)**	**0.097**	**-**	
NT-proBNP, per 100 pg/ml increase	**1.006 (1.003-1.009)**	**<0.001**	**1.004 (1.001-1.008)**	**0.007**
History of coronary artery disease	0.994 (0.506-1.951)	0.986		
History of atrial fibrillation	1.325 (0.761-2.304)	0.320		
History of hypertension	0.640 (0.346-1.184)	0.155		
History of diabetes	0.745 (0.335-1.658)	0.471		
History of hyperlipidemia	0.914 (0.457-1.830)	0.800		
Use of loop diuretic	**5.504 (1.298-23.331)**	**0.021**	**-**	
CO, l/min	**0.768 (0.619-0.952)**	**0.016**	**-**	
Heart rate, bpm	1.007 (0.986-1.027)	0.520		
MAP, mmHg	**0.968 (0.940-0.996)**	**0.027**	**-**	
RAP, mmHg	**1.072 (1.028-1.119)**	**0.001**	**1.067 (1.023-1.113)**	**0.002**
mPAP, mmHg	**1.048 (1.018-1.080)**	**0.002**	**-**	
PAWP, mmHg	**1.044 (0.997-1.093)**	**0.066**	**-**	
CPO_{RAP}, W	**0.102 (0.027-0.391)**	**0.001**	**0.211 (0.052-0.864)**	**0.030**

BMI indicates body mass index; CO, cardiac output; CPO_{RAP}, right atrial pressure-corrected cardiac power output; LVEF, left ventricular ejection fraction; NYHA, New York Heart Association; MAP, mean arterial pressure; mPAP, mean pulmonary arterial pressure; NT-proBNP, N-terminal pro-B-type natriuretic peptide; PAWP, pulmonary arterial wedge pressure; RAP, right atrial pressure; TAPSE, tricuspid annular plane systolic excursion.

Supplemental Table 2. Univariate and multivariate Cox regression models for event-free survival.

	Univariate Cox Regression		Multivariate Cox Regression	
	**HR (95%CI)**	**P value**	**HR (95%CI)**	**P value**
Age, y	0.992 (0.975-1.010)	0.396		
Sex, Male vs. Female	1.127 (0.646-1.967)	0.673		
BMI, kg/m^2	**0.928 (0.856-1.007)**	**0.074**	**-**	
NYHA functional class, Ⅳ vs. Ⅱ+Ⅲ	**2.062 (1.165-3.650)**	**0.013**	**-**	
LVEF, %	0.982 (0.951-1.014)	0.258		
TAPSE, mm	**0.948 (0.890-1.010)**	**0.097**	**-**	
NT-proBNP, per 100 pg/ml increase	**1.006 (1.003-1.009)**	**<0.001**	**1.005 (1.001-1.008)**	**0.007**
History of coronary artery disease	0.994 (0.506-1.951)	0.986		
History of atrial fibrillation	1.325 (0.761-2.304)	0.320		
History of hypertension	0.640 (0.346-1.184)	0.155		
History of diabetes	0.745 (0.335-1.658)	0.471		
History of hyperlipidemia	0.914 (0.457-1.830)	0.800		
Use of loop diuretic	**5.504 (1.298-23.331)**	**0.021**	**-**	
CO, l/min	**0.768 (0.619-0.952)**	**0.016**	**-**	
Heart rate, bpm	1.007 (0.986-1.027)	0.520		
MAP, mmHg	**0.968 (0.940-0.996)**	**0.027**	**-**	
RAP, mmHg	**1.072 (1.028-1.119)**	**0.001**	**1.081 (1.035-1.128)**	**<0.001**
mPAP, mmHg	**1.048 (1.018-1.080)**	**0.002**	**-**	
PAWP, mmHg	**1.044 (0.997-1.093)**	**0.066**	**-**	
CPO, W	**0.219 (0.075-0.644)**	**0.006**	**0.270 (0.083-0.880)**	**0.030**

BMI indicates body mass index; CO, cardiac output; CPO, cardiac power output; LVEF, left ventricular ejection fraction; NYHA, New York Heart Association; MAP, mean arterial pressure; mPAP, mean pulmonary arterial pressure; NT-proBNP, N-terminal pro-B-type natriuretic peptide; PAWP, pulmonary arterial wedge pressure; RAP, right atrial pressure; TAPSE, tricuspid annular plane systolic excursion.

Supplemental Figure 1

Kaplan–Meier estimates of time to event-free survival stratified by CPO_{RAP} for patients with pulmonary vascular resistance ≤ 2.2wood **(A)**, stratified by CPO_{RAP} for patients with pulmonary vascular resistance > 2.2wood **(B)**, stratified by CPO_{RAP} for patients with mean pulmonary arterial pressure ≤ 30mmHg **(C)**, stratified by CPO_{RAP} for patients with mean pulmonary arterial pressure > 30mmHg **(D)**.

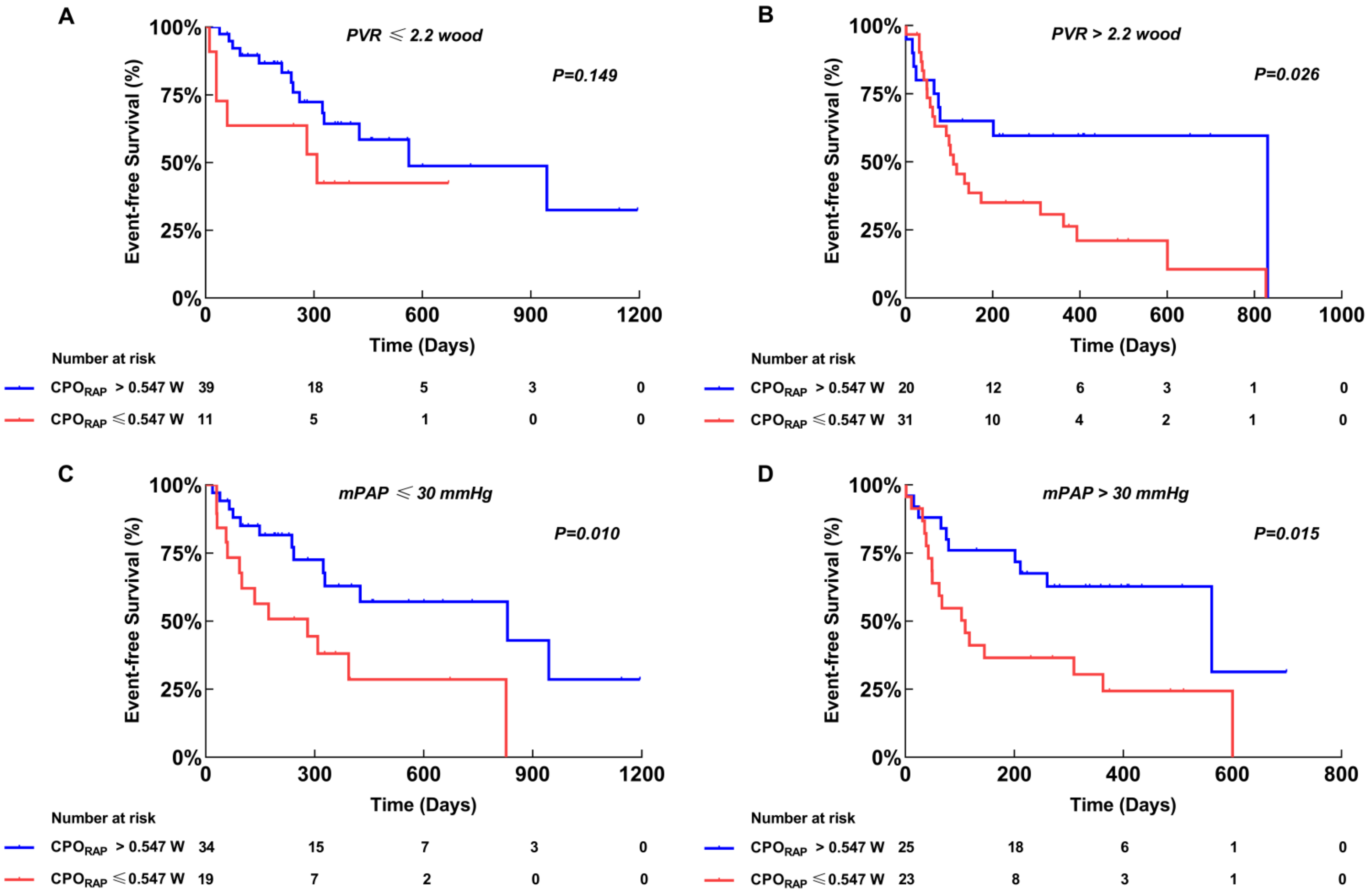
A
PVR ≤ 2.2 wood
P=0.149
100%
75%
50%
25%
0%
Event-free Survival (%)
0
300
600
900
1200
Time (Days)
Number at risk
CPORAP > 0.547 W 39 18 5 3 0
CPORAP ≤0.547 W 11 5 1 0 0
B
PVR > 2.2 wood
P=0.026
100%
75%
50%
25%
0%
Event-free Survival (%)
0
200
400
600
800
1000
Time (Days)
Number at risk
CPORAP > 0.547 W 20 12 6 3 1 0
CPORAP ≤0.547 W 31 10 4 2 1 0
C
mPAP ≤ 30 mmHg
P=0.010
100%
75%
50%
25%
0%
Event-free Survival (%)
0
300
600
900
1200
Time (Days)
Number at risk
CPORAP > 0.547 W 34 15 7 3 0
CPORAP ≤0.547 W 19 7 2 0 0
D
mPAP > 30 mmHg
P=0.015
100%
75%
50%
25%
0%
Event-free Survival (%)
0
200
400
600
800
Time (Days)
Number at risk
CPORAP > 0.547 W 25 18 6 1 0
CPORAP ≤0.547 W 23 8 3 1 0
